# Scoping Review: The Trajectory of Recovery of Participation Outcomes following Stroke

**DOI:** 10.1155/2018/5472018

**Published:** 2018-09-09

**Authors:** Batya Engel-Yeger, Tamara Tse, Naomi Josman, Carolyn Baum, Leeanne M. Carey

**Affiliations:** ^1^Department of Occupational Therapy, Faculty of Social Welfare and Health Sciences, University of Haifa, Haifa, Israel; ^2^Occupational Therapy, Department of Community and Clinical Allied Health, School of Allied Health, La Trobe University, Melbourne, VIC, Australia; ^3^Neurorehabilitation and Recovery, Stroke Division, The Florey Institute of Neuroscience and Mental Health, Heidelberg, VIC, Australia; ^4^Occupational Therapy Department, St Vincent's Hospital Melbourne, Fitzroy, VIC, Australia; ^5^Program in Occupational Therapy, Washington University School of Medicine, St Louis, MO, USA

## Abstract

Participation is a central concept in health and well-being and healthcare, yet operationalizing this concept has been difficult. Its definition, uses in healthcare, and impacts on recovery require ongoing research. Our review question goes like this: from the longitudinal evidence investigating participation among stroke survivors, what are the patterns of participation recovery in stroke survivors over time, and what interventions are used to improve participation? To fully understand these questions, we also ask, how is participation defined in the stroke literature, and what are the measures of participation used in the stroke literature? A systematic scoping review was undertaken using the search terms “stroke,” “longitudinal,” “participation,” and “outcome” in seven databases. Articles included were published until April 2017, written in English, and had at least two longitudinal assessments of participation. Fifty-nine articles met the inclusion criteria. The International Classification of Functioning, Disability and Health was the most frequent definition of participation used (34%). There were 22 different measures of participation. Eight of ten studies demonstrated significant improvements in participation up to 12 months poststroke. Efficacy of interventions and their impact on participation varied. The various definitions, measures, and intervention efficacies of participation highlight the need for further research worldwide into achieving meaningful participation and quality of life among stroke survivors. Future practice should include participation as a main outcome measure.

## 1. Introduction

Stroke is the leading cause of adult disability worldwide [1]. Stroke remains a major global health concern, and its significance is likely to increase in the future due to ongoing demographic changes including the aging of the population and health transitions [2, 3].

Participation is considered a major outcome of successful rehabilitation [4–6] and an essential component of rehabilitation science [7]. Previous findings suggest that participation is a concern for stroke survivors [4], is considered an unmet need [8], is influenced by the environment [9], and may be affected by age, acceptance of stroke, body functions (including upper limb function, depression, and other comorbidities [10, 11]), cognition [12], skills like walking, and stroke severity [13].

Stroke is a chronic condition for survivors, with long-term implications such as loss of control over their bodies, valued activities, meaningful skills, and social roles [14, 15] which may disrupt their daily life, relationships, and expectations of the future [16]. These multiple losses may further influence one's ability to participate in everyday life activities across their lifespan, thus highlighting the importance of investigating participation outcomes among stroke survivors over an extended period of time [17]. While task-specific and learning-based approaches to rehabilitation have the strongest evidence base [18], evidence regarding participation after stroke and intervention programs for enhancing participation in the long term is lacking [19]. Moreover, rehabilitation studies do not often include participation outcomes [19], and studies that do refer to participation do not often use a conceptual framework nor a clear definition of participation. This lack of consensus surrounding the conceptualization of participation has led to difficulties operationalizing participation [20–23]. These difficulties may result from the diverse definitions and interpretations of participation as a concept and from the wide variety of tools purporting to measure participation [24], making participation evaluation variable, challenging, and difficult to interpret.

In summary, participation is a central concept in healthcare and in disciplines such as occupational therapy [25]. Yet its definition and inclusion in health outcomes and its impacts on recovery over time are relatively limited to date and require ongoing research [7]. The rising prevalence of stroke and its significant consequences, in particular, the fact that participation is a significant factor that affects people's functioning [26], emphasize that it is essential to better understand the recovery of participation as an outcome and how participation may be a targeted outcome in interventions for stroke survivors. This directed investigation may contribute to the conceptualization of participation and its application in health theory and practice [25].

### 1.1. Objective of the Scoping Review

To the best of our knowledge, a scoping review of the literature investigating the recovery of participation outcomes after stroke has not been conducted. The aim of this scoping review was to critically review the evidence investigating recovery of participation outcomes following stroke. The main questions guiding our review evaluation and evidence synthesis of the longitudinal studies investigating participation after stroke: (i) what are the patterns of recovery in participation outcomes in stroke survivors over time and (ii) what interventions are used to improve participation? To fully understand these questions, we also ask, how is participation defined, and what are the measures of participation used in the stroke literature?

## 2. Materials and Methods

This scoping review was based on the methods outlined by Arksey and O'Malley [27], which include six iterative steps: (1) identifying the research question; (2) searching for relevant studies; (3) selecting the studies; (4) charting the data; (5) collating, summarizing, and reporting the results; and (6) consulting with stakeholders to inform or validate findings. A scoping review methodology was selected because it can include broad questions and a range of research approaches surrounding a topic of interest. This methodology assists to identify the gaps in the current knowledge base to help guide future research in the field. Step 6, consultation with stakeholders is optional. We did not directly consult stroke stakeholders. However, ongoing consultation by the authors as the key stakeholders occurred throughout the review process.

The research question and the search terms were developed in consultation amongst the authors. The search terms were related to the study population, the intervention, the comparison or outcome, and the types of study design to include in the review. Seven databases were searched: EMBASE, PubMed, Web of Science, CINAHL, CINAHL Plus, Medline, and PsycINFO using the search terms “stroke,” “longitudinal,” “participation,” and “outcome.” Synonyms, wildcards, and Boolean operators were used in the search strategy (Table 1). Study designs included were longitudinal cohort, case control, pre-post test, and case series and case report studies with or without intervention. Included studies were written in English, published up to April 2017, and had at least two participation evaluation time points in the same sample, and with the same participation instrument, as defined by the authors of the study under review. Studies investigating paediatric stroke and severe comorbidities such as Alzheimer's, diabetes, and cancer were excluded.

### 2.1. Data Extraction

Three reviewers worked together to evaluate all articles for this review using Covidence online systematic review platform [28]. Each article was independently reviewed following a systematic process according to the inclusion and exclusion criteria. Any disagreements between reviewers were resolved by consensus.

## 3. Results

**Figure 1:**
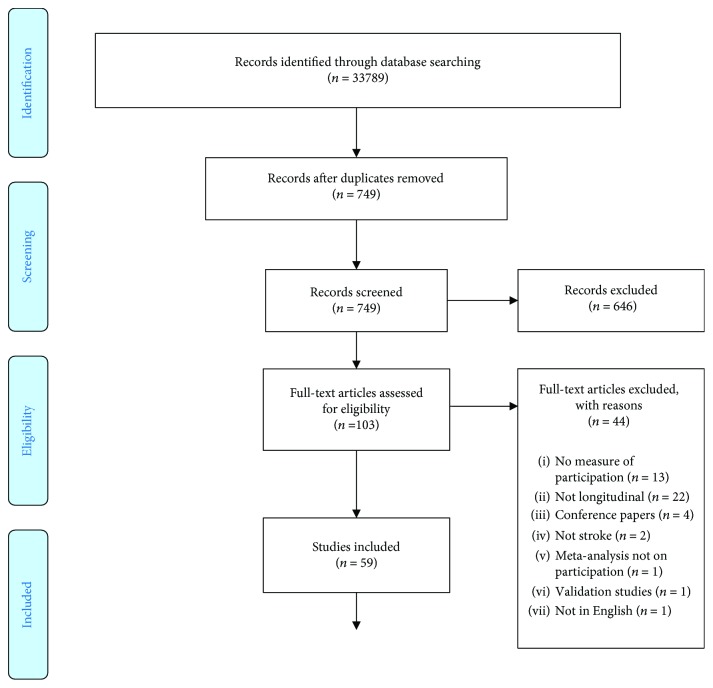
PRISMA 2009 flow diagram.

The flow of studies through the process is shown in Figure 1. The final number of studies included in this scoping review was 59. The summary of data extracted from each of the articles is provided in Table 2. Most of the studies included an assessment of participation in a community setting (85%); four of the 59 studies (7%) included assessments of participation only in an inpatient setting; and three studies did not state the setting location. Sixteen studies did not describe the assessor; of the remaining studies, the majority (81%) of assessors were physiotherapists and occupational therapists. When grouped into continents, the majority of the studies were based in North America (47%), followed by Europe (32%), Australasia (15%), Africa (3%), mixed countries (3%), and South America (2%). Interestingly, the earliest study in our scoping review was in 2001.

### 3.1. Patterns of Participation Recovery after Stroke

Of the 59 studies, all included two time points, 38 had a third measurement time of participation, 18 had a fourth, and 18 had a fifth measurement time. The terminology used to describe when participation was measured varied across the studies. Thirty-four of the studies (58%) called the first measure a baseline measure; the remaining studies described the measure in terms of a time point poststroke (37%) or postintervention/discharge (5%). The most frequent measurements of participation poststroke were 6 months, then 3 months, and then 12 months (see Table 3 for details).

Following an intervention (35 of the 59 studies), the most frequent time to measure participation was immediately after the intervention (32%). The interventions ranged in duration (e.g., 30 hours of therapy to 4 months of therapy). The next most frequent time point to measure participation following an intervention was 6 months, followed by 3 months postintervention. Four studies measured participation following a period after discharge from a hospital/rehabilitation unit or physiotherapy. One study did not specify whether the 12-month follow-up was 12 months after baseline, intervention, or poststroke.

Although all 59 studies reported at least two measurement times of participation after stroke, only 10 studies statistically tested for change during the natural recovery of participation over time. Of these 10 studies, 8 demonstrated a significant improvement in participation over time. These eight studies included the following time points: stroke to 3 months; stroke to 6 months; 2-3 months to 6 months; and 6 months to 12 months. The two studies that did not find a significant change included one study that tested participation at a mean time poststroke of 6 years poststroke and then measured participation again 3 months later following intervention. The other study did not show a significant improvement from 3 months to 6 months poststroke.

### 3.2. Intervention Efficacy and Impact on Participation

There were 17 randomized control trials included in this review, as detailed in Table 4. Of the 12 studies, 8 demonstrated a significant association with participation. Three of these studies used a form of supervised exercise program, compared to usual care, to improve participation, and measured using the Participation domain of the Stroke Impact Scale (SIS-P). One study demonstrated the use of a leisure therapy program on improved participation, measured in minutes engaged in leisure activities and the number of leisure activities compared to controls. One study showed that the use of therapist-supervised repetitive task practice (RTP) had a greater effect on participation than RTP combined with robotic-assisted therapy at 2 months follow-up. Three studies found that participation improved over time regardless of the intervention (cognitive behavioral therapy versus computerized cognitive training, aerobic exercise versus no therapy, and patient education program versus placebo group).

The four studies that did not demonstrate a significant relationship with participation included three interventions focusing on the use of specific physical therapy interventions (foot drop stimulator versus standard ankle foot orthosis, body weight–supported exercise compared to overground walking training, and community-based fitness and mobility exercise protocol versus usual care) and one intervention focusing on a client-centred activities of daily living (ADL) program versus usual care.

### 3.3. Measuring Participation

There were 22 different measures of participation used in the included studies. The SIS-P was the tool used by 24 of 59 studies (46%) included in this review, as detailed in Table 5. Of the 24 studies that used the SIS-P, 9 used the ICF definition of participation, 13 used an operational definition, four used “meaningful activities/occupations,” two used “community participation,” and one used the term “social participation.” The next most frequent measure of participation was the LIFE-H. All studies using the LIFE-H (*n* = 5) used the Disability Creation Process conceptual framework definition. Four studies used the London Handicap Scale; of these, three used the International Classification of Functioning, Disability and Health (ICF) definition of participation, and the other used an operational definition. Three studies used the Utrecht Scale for Evaluation of Rehabilitation-Participation; of these, all used an operational definition of participation. Three studies used the Short Form Health Survey (SF-36); of these, two used the operational definition, and the other used the term “role participation.”

### 3.4. Definitions of Participation

Of the 59 studies included in this review, many did not provide a definition of participation (41%), instead only describing the tool used in the study as measuring participation (e.g., “participation was measured using the Stroke Impact Scale”). This was categorized as an operational definition. Of the remaining studies, the most frequent definition of participation was the ICF definition (34%), “i.e. involvement in a life situation.” The remaining definitions used by two or fewer studies are reported in Table 6.

When we compared the definition of participation used in the study as a proportion of the studies from each of the continents, we found that operational definitions and the ICF definition were widely used across all continents (see Table 7).

## 4. Discussion

This scoping review aimed to critically review the evidence regarding patterns of recovery of participation outcomes among stroke survivors and to summarize the patterns of recovery and intervention efficacy on participation outcomes over time. The earliest publication included in this scoping review was in 2001, when the World Health Organization (WHO) endorsed the ICF, of which participation is a core component, suggesting that the use of the term “participation” is related to the release of the ICF by the WHO. The impact of the ICF on participation may also be reflected by the origin of the included publications. Our scoping review revealed that the majority of the studies were conducted in North America—the origin of conceptual frameworks including participation such as the Person-Environment-Occupation-Performance (PEOP) and ICF [29]. Interestingly, this scoping review also included studies performed in many other counties and continents (e.g., Europe, Australasia, Africa, and South America), supporting the perception that participation is a major outcome measure of intervention and recovery and is accepted worldwide.

### 4.1. Patterns of Participation Recovery Outcomes over Time

The findings from this scoping review revealed that participation is most often measured 6 months poststroke, followed by 3 months poststroke, and 12 months poststroke. These findings may lead us to suggest that participation recovery occurs at these time points. However, this may not be the true trajectory of recovery of participation. Rather, we are limited by the measurement tools and time points under which they occurred. Nonetheless, previous studies have suggested that, among stroke survivors, progressive and significant functional recovery in participation outcomes may occur during the first 6 months [30]. The findings from our scoping review extends this knowledge, highlighting that improvements in participation does occur over time and up to 12 months poststroke. However, the percentage of the studies that performed these longer follow-ups to 12 months is low. There were even fewer studies conducting follow-up beyond 12 months. This may be due to the difficulties of a cohort study, such as the financial cost of conducting long-term studies, participant drop-outs, difficulties following up participants in rural and remote settings, and educational background of the population (the ability to read and write) [30].

### 4.2. Intervention Efficacy on Participation and Recovery

Findings of intervention efficacy and impact on participation were not consistent in the studies included in this scoping review—only some studies found improvement in participation resulting from posttreatment recovery. Some reported improvement in participation due to spontaneous recovery. Other studies did not find a relationship between intervention and participation.

The studies that found improvement in participation used varying intervention strategies, such as supervised exercise programs, leisure therapy programs, and repetitive task practice. The studies that did not find a relationship between intervention and participation applied specific techniques such as cognitive behavior therapy or focused on improving specific body functions, mainly motor functions (using, for example, foot drop stimulator, body weight support, or walking training). These results raise questions regarding the literature claiming that intervention should aim to improve one daily activity, such as walking, to enhance participation. As previous research has stressed [30], improvements in participation levels of patients with stroke require particular attention to situations demanding community, social, and civic involvement. Further, in this scoping review, several of the outcomes on participation referred to mobility, fitness, and other aspects of physical/motor function. It may be assumed that because these studies were performed by physiotherapists, special attention was given to this area. This supports Kjellberg et al. [31], who stated that participation in the physical field is highly represented in the literature of stroke survivors. To fully utilize and apply these findings in health theory and practice, they should be interpreted in relation to how the measurement of participation was conceptualized and measured by the studies in this scoping review.

### 4.3. Measuring Participation

This scoping review found various measures of participation that were used across studies. The most prevalent measures found in this scoping review were the SIS-P, followed by the LIFE-H. Previous studies investigating these tools and other tools purporting to measure participation have highlighted that the different tools measure different domains of participation (e.g., Community, Social and Civic Life, Domestic Life, and Activities of Daily Living) and different aspects of participation (i.e., frequency, restrictions, satisfaction); the administration and response formats are different (e.g., self-report, interviewer-administered), and the psychometric properties varied [24, 32–34]. For example, in the study by Tse et al. [24], the Participation domain of the SIS covered four of the nine Activities and Participation domains of the ICF, whereas the LIFE-H covered seven of the nine domains. Further, each tool covered each domain of the ICF to varying degrees: the SIS-P contained three items in the Community, Social and Civic Life domain of the ICF, whereas the LIFE-H contained nine. These differences in how participation is measured impacts on our future understanding and conceptualization of participation. For example, Kossi et al. [35], who measured participation using the Participation Measurement Scale (PM-Scale) that covers all nine ICF domains, found that some participation domains are affected by stroke more than others: participation in community, social, and civic life; interpersonal interactions and relationships; and domestic life [35]. Similarly, Heinemann et al. [36] stressed that greater restrictions in participation among stroke survivors are related to community, social, and civic life.

Further, it has been shown that the different aspects of participation are only partially correlated [37]. Blomer et al. [37] compared the association between participation frequency, participation restriction, and participation satisfaction using the Utrecht Scale for Evaluation of Rehabilitation-Participation. They found that the strongest independent association was between participation restriction and participation frequency in vocational activities. Participation frequency in leisure and social activities was not independently associated with participation restriction, nor was participation frequency in leisure and social activities associated with participation satisfaction. This finding suggests the need for measures of participation to cover the varying aspects of participation in discrete scores and not measures that combine aspects of participation into one overall score. We suggest that, because the SIS-P covers a brief range of domains in Activities and Participation section of the ICF and it combines different aspects of participation into one score, it is best described as a screening tool of participation.

### 4.4. Definitions of Participation

Since the publication of the ICF in 2001, the concept of participation has become central in discussions across rehabilitation science [5]. Yet this scoping review found that many publications did not provide a definition of participation but rather described the tool used in the study to measure participation (such as the SIS). The studies that used a definition of participation used varying definitions, such as role participation, community participation, social participation, participation as reflected in meaningful activities/occupations, or life habits. Nevertheless, the most frequently used definition was that of the ICF, which emphasizes that health is broader than a purely medical or biological conceptualization of dysfunction and must consider the influence of the environment and other contextual factors on functioning. Participation is defined by the ICF as an individual's involvement in life situations [26]. It represents the societal perspective of functioning. According to the ICF, functioning is the interaction of individuals with their physical, social, and environment. More concretely, emphasis is on the individual's ability to perform activities and to participate in real-life, everyday situations [26]. Indeed, since the publication of the ICF in 2001, the concept of participation has become central in discussions across rehabilitation science and practice.

Although the ICF conceptualization of participation is widely used, there are other conceptualizations of participation used within the health rehabilitation literature. The Person-Environment-Occupation-Performance (PEOP) is a model stemming from occupational therapy [29]. In the PEOP model, participation is defined as active engagement in daily life, families, work, and communities. In this model, occupational performance and participation are a result of the interaction between factors related to the person, the environment, and one's chosen activity or occupation. According to the PEOP model, occupational performance reflects the doing, and participation reflects the active engagement in life. The conceptual framework on participation by Hammel and colleagues' emphasizes the importance of participation choice, control, and engagement [38].

Using conceptual frameworks such as the ICF and the PEOP assist to develop theory and provide the rationale and guide the application of theory into practice [39]. The studies in this review conducted in Africa used only the ICF definition of participation, while those originating from other countries out of Africa (as seen in Table 7) used a variety of definitions of participation. Indeed, participation, specifically meaningful participation in everyday occupations, is a complex phenomenon to conceptualize and measure [40]. The reason for choosing one definition over another requires further study—is it because conceptual frameworks such as the ICF and the PEOP are not applied in specific geographic areas? Does it result from cultural reasons, from practical reasons such as the setting, or is it linked with existing evaluations of participation that cover definitions such as that of the ICF? Is it easier/more practical to measure participation in that specific definition? Are there financial reasons? Answers to these questions may help in establishing future studies and in turn better outcomes for stroke survivors.

Another finding is that the number of publications per year has not increased linearly. Considering that participation is an important outcome measure of intervention, it would be expected that the number of publications should rise. Research and practice should elucidate factors that may lead to an increase in participation outcomes: for example, what may enhance the conduct of studies investigating participation in stroke survivors and longitudinal studies relating to intervention efficacy on participation? This information may contribute to evidence-based practice for the benefit of stroke survivors expressed in better engagement in real-life settings, meaningful participation, and better quality of life.

*To summarize*, participation is a critical factor that should be considered in intervention programs for stroke survivors. The various definitions of participation, the assessments, and the limited information about intervention efficacy in meanings of participation highlight that further studies should be performed worldwide and contribute to a coherent and consistent discussion targeted at achieving meaningful participation among stroke survivors.

Considering the challenges that stroke survivors face and that participation is a critical outcome measure of intervention, evaluations of participation should reflect meaningful participation—the subjective experience of the individual's performance of activities [41], the enjoyment from participating in the activity [42–44], the context where the participation takes place, and also the activities desired by the individual.

### 4.5. Implications for Stroke Rehabilitation

Participation as a main outcome measure of intervention should continue to receive special attention in rehabilitation programs for stroke survivors. For example, occupational therapy intervention programs for participation should include clinical reasoning, in which therapists profile the individual's challenges, map problem priorities, and, together with the individual, set meaningful goals to enhance participation in real-life context to achieve the optimal rehabilitation experience. As such, therapists should combine self-reports with observations, use an elaborated point of view to understand factors that influence participation (including personal and environmental factors), and use conceptual models such as the PEOP alongside theoretical frameworks such as the ICF to accurately understand these complicated relationships [45] and focus interventions accordingly. Therapists must also consider the measurement tool used to assess participation. Different tools assess different domains and aspects of participation [24]. Consistent use of the most appropriate participation measure will assist to meet stroke survivor's specific participation needs.

### 4.6. Implications for Research

In general, further studies are needed in order to (1) profile participation among stroke survivors as an outcome measure of recovery and/or intervention and (2) expand the body of knowledge about study designs, sensitive assessments, and time points of evaluations that may provide data about occupation-based interventions and their effectiveness in terms of participation and well-being.

More studies should be performed by disciplines where participation is the focus, such as occupational therapy, and we must extend beyond the emphasis found today on motor function and mobility; provide more data about the interaction between body function, performance, and participation; illuminate the interaction between personal and environmental factors; and consider contextual factors such as sociocultural background to find optimal strategies that meet patients' specific needs and interests.

#### 4.6.1. Strengths and Limitations

Strengths of this review include using recommended and rigorous methods widely accepted in the conduct of scoping reviews and using broad search terms across a range of databases in order to maximize the likelihood of capturing the available research in the recovery of participation outcomes following a stroke. Limitations of this scoping review result from the variability in studies' designs and methods, their definitions of participation, the relatively small number of studies that examine intervention impacts on participation in stroke survivors, and the multiple assessments, assessors, and interventions, which make it difficult to profile the effects of specific intervention tools and strategies on participation. Many studies focused on symptom management and on activities of daily living. Participation evaluation mainly referred to type of activities and did not use an elaborated perspective about where and with whom does the individual participate and how much they enjoy engaging in the activity. Further studies focussing on participation outcomes may contribute to filling this gap in research.

## 5. Conclusion

Stroke rehabilitation research and practice regarding stroke survivors should refer to participation as a major outcome measure of recovery and intervention effectiveness. Assessments should be used that include a broad perspective on participation domains. However, tools measuring participation must not combine the different aspects of participation into one overall score. This will assist us to better understand which interventions have a better impact on participation and recovery.

Further research should be performed to support occupation-based intervention effectiveness for providing stroke survivors optimal intervention, meaningful participation, and meaningful life.

## Figures and Tables

**Table 1:** Search terms.

cerebrovascular	and	participation.sh. OR	and	longitudinal study.sh. OR	and	outcome
accident.sh. OR stroke.ti.		participation.ti. OR		longitudinal stud^∗^.ti. OR		assessment.sh. OR
OR stroke.ab. OR cerebro		participation.ab. OR patient		longitudinal stud^∗^.ab. OR		outcome
vascular accident.ti. OR		participation.sh. OR social		longitudinal eval^∗^.ti. OR		measurement.sh.
cerebro vascular		participation.sh. OR patient		longitudinal eval^∗^.ab. OR		OR patient outcome
accident.ab. OR cerebral		involvement.ti. OR patient		longitudinal survey.ti. OR		assessment.sh. OR
vascular accident.ti. OR		invovlement.ab. OR community		longitudinal survey.ab. OR		treatment
cerebral vascular		participation.ti. OR community		prospective stud^∗^.ti. OR		outcome.sh. OR
accident.ab. OR brain		participation.ab. OR community		prospective stud^∗^.ab. OR		outcome^∗^.ti. OR
ischaemic attack.ti. OR		integration.ab. OR community		follow up.sh. OR follow^∗^ up.ti.		outcome^∗^.ab. OR
brain ischaemic attack.ab.		integration.ti. OR client		OR follow^∗^ up.ab. OR follow		measure^∗^.ti. OR
OR brain ischemic attack.ti.		participation.ab OR client		up stud^∗^.ti. OR follow up		measure^∗^.ab. OR
OR brain ischemic		participation.ti. OR social		stud^∗^.ab.		asses^∗^.ti. OR
attack.ab. OR brain		integration.ab. OR social				asses^∗^.ab. OR
vascular accident.ti. OR		integration.ti. OR community				eval^∗^.ti. OR
brain vascular accident.ab.		involvement.ab OR community				eval^∗^.ab.
OR CVA.ti. OR CVA.ab. OR		invovlement.ti. OR activity				
ischaemic cerebral		participation.ab OR activity				
attack.ti. OR ischaemic		participation.ti				
cerebral attack.ab. OR						
ischemic cerebral attack.ti.						
OR ischemic cerebral attack.ab.						

**Table 2:** Extracted data from studies included in the scoping review on longitudinal participation outcomes after stroke.

Author	Year	Country	Setting	Study design	Measure	Sample size	1^st^ measure	2^nd^ measure	3^rd^ measure	4^th^ measure	Assessors	Age (*yr*) mean (SD), median (IQR)	Define participation
Altman et al. [46]	2013	USA	Community	Retrospective cohort of completers and noncompleters	MPAI-4	Completers *n* = 738, noncompleters *n* = 150	Baseline	Discharge	Postdischarge (3 months)	Postdischarge (12 months)	Not described	Completers 51.10 (11.46), noncompleters 52.96 (52.96)	Operational
Awad et al. [47]	2014	USA	Research laboratory	Case series pretest, posttest	SIS-P	*n* = 13	Baseline	Postbaseline (12 weeks)			PT	61 (8.31)	Self-perceived participation
Beaudoin et al. [48]	2013	Canada	Community	Prospective cohort study	LIFE-H	*n* = 57	Baseline	Postbaseline (6 months)	Postbaseline (9 months)		Not described	76.9 (8.1)	DCP
Bertilsson et al. [49]	2016	Sweden	Inpatient and community	Multicentre cluster RCT	SIS-P, OGQ	Client-centred *n* = 88, usual *n* = 95	Baseline	Postbaseline (3 months)	12 months		OT	Client-centred 74.1 (9.5), usual 71.3 (10.1)	ICF, meaningful activities/occupation
Brown et al. [50]	2014	Canada	Community	Prospective cohort study, with intervention and interrupted time series	PASIPD	*n* = 61	Baseline	Postbaseline (2 months)	Postbaseline (4 months)		PT	Intervention 65 (13), control 66 (13)	Operational
Butler et al. [51]	2006	USA	Community	Case study: pre-post test	SIS-P	*n* = 1	Baseline	Postbaseline (4 weeks)	Postintervention (8 weeks)	Postintervention (3 months)	OT	44	ICF
Chou et al. [52]	2015	Taiwan	Inpatient and community	Prospective cohort study	SIS-P	Baseline *n* = 263	Baseline	Postbaseline (2 weeks)			OT	59.8 (13.0)	Operational
Combs-Miller et al. [53]	2014	USA	Research laboratory and community	RCT	IMPACT-P	*n* = 20	Baseline	Postintervention	Postbaseline (3 months)		PT	Body weight-supported 56.20 (7.61), overground walking 65.50 (6.17)	ICF
Demetrios et al. [54]	2014	Australia	Community	Nonrandomized controlled study	GAS	High-intensity program *n* = 28, usual care *n* = 31	Baseline	Postintervention (6 weeks)	Postintervention (12 weeks)	Postintervention (24 weeks)	Not described	High-intensity 60.6 (48.6–65.9), usual care 61.4 (47.8–68.6)	ICF
Desrosiers et al. [55]	2006	Canada	Community	Prospective cohort study	LIFE-H	T1 *n* = 102, T2 *n* = 66	Poststroke (6 months)	Poststroke (2–4 years)			OT	T1 68.1 (14.1), T2 67.6 (13.7)	DCP
Desrosiers et al. [56]	2007	Canada	Community	RCT	Minutes	Experimental *n* = 29, control *n* = 27	Baseline	Postintervention			OT	Experimental 70.0 (10.2), control 70.0 (12.0)	DCP
Desrosiers et al. [57]	2006	Canada	Community	Prospective cohort study	LIFE-H	T1 *n* = 102, T2 *n* = 66	Postdischarge (6 months)	Poststroke (2–4 years)			OT	T1 68.1 (14.1), T2 67.6 (13.7)	DCP
Egan et al. [58]	2014	Canada	Community	Prospective cohort study	RNL	*n* = 67	Poststroke (6 months)	Poststroke (9 months)	Poststroke (12 months)	Poststroke (18 months)	Not described	64.8 (13.3)	ICF
Egan et al. [59]	2015	Canada	Community	Prospective cohort study	RNL	*n* = 67	Poststroke (6 months)	Poststroke (9 months)	Poststroke (12 months)	Poststroke (18 months)	Not described	64.8 (13.3)	ICF
Evan et al. [60]	2012	USA	Community	Case study pre-post test	GPS	*n* = 1	Baseline	Postbaseline (4 weeks)	Postbaseline (8 weeks)	Postdischarge (6 and 12 months)	PT	56	ICF
Flansbjer et al. [61]	2012	Sweden	Community	Prospective cohort study follow-up from RCT	SIS-P	*n* = 18	Baseline	Postintervention	Postintervention (5 months)	Postintervention (4 years)	PT	4 years 66 (4)	ICF
Flansbjer et al. [62]	2008	Sweden	Community	RCT	SIS-P	*n* = 24	Baseline	Postintervention	Postintervention (5 months)		PT	Intervention 61 (5), control 60 (5)	ICF
Guidetti et al. [63]	2015	Stockholm, Uppsala, and Gävleborg County, Sweden	Community	RCT	SIS-P	*n* = 280	Poststroke (3 months)	Poststroke (6 months)	Poststroke (12 months)		OT	CADL 74 (10), UADL 71 (11)	Operational
Guidetti et al. [64]^∗^	2014	Stockholm, Sweden	Community	Comparative study no controls	SIS-P	*n* = 349	Poststroke (3 months)	Poststroke (12 months)			OT, PT	74 (14)	Operational
Hamzat and Peters [65]^∗^	2009	Nigeria	Community	Longitudinal descriptive study	LHS	*n* = 20	Poststroke (1 month)	Poststroke (2 months)	Poststroke (3 months)	Poststroke (4 months)	PT	Not reported	ICF
Horgan et al. [66]^∗^	2009	Ireland	Community	Comparative study no controls	FAI	*n* = 23	Poststroke (2 weeks)	Poststroke (6 months)	Poststroke (12 months)		PT	69.7 (11.3)	Operational
Ilse et al. [67]	2008	Belgium	Community	Comparative study no controls	NEADL, mRS	*n* = 90	Poststroke (2 months)	Poststroke (4 months)	Poststroke (6 months)		PT	67.3 (11.2)	Operational
Baert et al. [68]	2012	Belgium	Inpatient	Case series pretest, posttest	SIS-P, NEADL	*n* = 50	Baseline	Poststroke (2 months)	Poststroke (6 months)	Poststroke (12 months)	PT	57.2 (11.4)	ICF
Jalayondeja et al. [69]	2011	Thailand	Community	Prospective cohort study	SIS-P	*n* = 98	Poststroke (1 month)	Poststroke (3 months)	Poststroke (6 months)		Researcher/research assistant	61.9 (11.02)	Operational
Jalayondeja et al. [70]^∗^	2014	Thailand	Community	Prospective cohort study	SIS-P	*n* = 98	Poststroke (1 month)	Poststroke (3 months)	Poststroke (6 months)		Researcher/research assistant	Fallers 65.4 (10.2), nonfallers 60.7 (11.2)	Community participation
Kluding et al. [71]	2013	USA	Community	RCT	SIS-P	*n* = 197	Baseline	Postintervention (6 weeks)	Postintervention (12 weeks)	Postintervention (30 weeks)	PT	Interventions 60.7 (12.2), control 61.6 (11.0)	Operational
Kootker et al. [72]	2017	Netherlands	Community	RCT	USER-P	*n* = 61	Baseline	Postintervention	Postintervention (4 months)	Postintervention (8 months)	Health psychologist	CBT 61 (45–79), CCT 61 (25–76)	Operational
Kutner et al. [73]	2010	USA	Inpatient and community	RCT	SIS-P	RTP *n* = 7, combined therapy group *n* = 10	Baseline	Postintervention	Postintervention (2 months)		OT, PT	RTP 51.0 (11.3), combined therapy group 61.9 (13.4)	Operational
Kwok et al. [74]^∗^	2011	Hong Kong	Inpatient	Observational cohort study	LHS	Baseline *n* = 594, 3 months *n* = 500, 12 months *n* = 433	Poststroke (3 months)	Poststroke (12 months)			Not described	72 (65–77)	ICF
Laufer et al. [75]	2009	Israel	Research laboratory and community	Time series no control	SIS-P	*n* = 24	Baseline	Postintervention (8 weeks)	Postintervention (12 months)		PT	Study group 55.0 (14.6)	Meaningful activities/occupation
Lennon et al. [76]	2006	Ireland	Inpatient and community	Pre-post test	LHS	*n* = 9	Poststroke (6 weeks)	Discharge from physiotherapy				64.3 (9.6)	Operational
Levin et al. [77]	2012	Canada + Israel	Research laboratory and community	Case series pre-post test	MAL	VR *n* = 8, conventional *n* = 6	Baseline	Postbaseline (1 week)	Postintervention	Postintervention (1 month)	OT	VR 58.1 (14.6), conventional 59.8 (15.1)	ICF
Lund et al. [78]	2012	Norway	Community	RCT	SF-36	Intervention *n* = 39, control *n* = 47	Poststroke (3 months)	Poststroke (9 months)			Researcher/research assistant	Intervention 75 (7.2), control 79 (6.5)	Operational
Mayer and Reid [79]^∗^	2004	Canada	Community	Prospective longitudinal cohort study	IPA	*n* = 18	Poststroke (3 months)	Poststroke (6 months)			OT	67.4 (13.4)	ICF
Mayo et al. [80]	2009	Canada and England	Inpatient and community	Longitudinal cohort study	SIS-P	*n* = 408	Poststroke (1 month)	Poststroke (3 months)	Poststroke (6 months)	Poststroke (12 months)	Not described	66.5 (14.6)	Operational
Mayo et al. [81]	2011	Canada	Community	Reanalysis of RCT	SF-36	Nurse case-manager group *n* = 96, usual care group *n* = 94	Postintervention	Postintervention (6 months)			Not described	Nurse case-manager 70 (14), usual care 72 (13)	Role participation
Mayo et al. [82]	2013	Canada	Community	RCT	SIS-P, RAND-36	Cycle group *n* = 43, exercise group *n* = 44	Baseline	Postbaseline (12 months)			PT	Cycle 67.7 (14.4) ,exercise 67.8 (12.3)	ICF, role participation
Mercer et al. [83]	2009	USA	Inpatient and research laboratory	Prospective cohort study	SIS-P	*n* = 33	Poststroke (1 month)	Poststroke (3 months)	Poststroke (6 months)		Not described	58.7 (17.3)	ICF
Nijenhuis et al. [84]	2015	Netherlands	Community	Comparison within subjects, longitudinal	SIS-P	*n* = 24	Baseline	Postintervention (6 weeks)			Biomechanical engineering	59 (13)	Operational
Page et al. [85]	2015	USA	Research laboratory and community	Comparison within subjects longitudinal no control	SIS-P	*n* = 5	Baseline	Postintervention			OT	43.7 (6.43)	Meaningful activities/occupation
Pang et al. [86]	2005	Canada	Community	RCT	PASIPD	*n* = 63	Baseline	Postintervention			OT, PT	Intervention group 65.8 (9.1), control 64.7 (8.4)	Operational
Parker et al. [87]	2001	UK	Community	RCT	NLQ	*n* = 466	Postintervention (6 months)	Postintervention (12 months)			OT	Leisure 72 (65–79), ADL 71 (66–78), control 72 (65–78)	Operational
Penney et al. [88]	2007	Canada	Not stated	Single case study	IPA, 6-minute walk test	*n* = 1	Poststroke (3 months)	Poststroke (12 months)			PT	62	ICF
Pundik et al. [89]	2012	USA	Not stated	Pre-post test with interrupted time series no control	SIS-P	*n* = 44	Baseline	Postintervention	Postintervention (3 months)		Not described	60 (16.8)	ICF
Raghavan et al. [90]	2016	USA	Community	Mixed-method pre-post design with 1 year follow-up	SIS-P	*n* = 13	Baseline	Postintervention	Postintervention (12 months)		OT	52 (14)	Operational
Rochette et al. [91]	2013	Canada	Inpatient and community	RCT	LIFE-H	*n* = 186	Baseline	Postbaseline (6 months)	Postintervention (12 months)		OT, PT	YOU CALL 63.2 (12.4), WE CALL 61.7 (12.7)	DCP
Sabariego et al. [92]	2013	Germany	Inpatient and community	RCT	SIS-P	*n* = 260	Baseline	Postintervention (1 week)	Postintervention (6 months)		Not described	Experimental 55.3 (12.6), control 59.3 (12.7)	ICF
Sandberg et al. [93]	2016	Sweden	Community	RCT	SIS-P	*n* = 56	Baseline	Postintervention (3 months)			Not described	Intervention 71.3 (7.0), control 70.4 (8.1)	Social participation
Segura et al. [94]	2006	Brazil	Research laboratory and community	Prospective, comparative, no control	SIS-P	*n* = 18	Baseline	Postintervention (3 months)			PT	52.9	Operational
Singam et al. [95]	2015	Sweden	Inpatient	Prospective, longitudinal study	FAI	*n* = 349	Poststroke (5 days)	Poststroke (3 months)	Poststroke (6 months)	Poststroke (12 months)	OT, PT	69.4 (13.8)	ICF
Stuart et al. [96]	2009	Italy	Not stated	Nonrandomized control trial	SIS-P	Intervention *n* = 40, control *n* = 38	Baseline	Postintervention (6 months)			Not described	Intervention 66.8 (1.4), control 70.0 (1.7)	Operational
Studenski et al. [97]	2005	USA	Community	RCT	SIS-P	Intervention *n* = 44, usual care *n* = 49	Baseline	Postintervention	Postintervention (6 months)		Blinded assessor	Intervention 68.5 (9.0), usual care 70.4 (11.3)	Operational
Teoh et al. [98]^∗^	2009	Australia	Community	Longitudinal cohort study	SIS-P	*n* = 135	Baseline	Postbaseline (10 weeks)	Postbaseline (6 months)		Not described	67.5 (14.3)	Social participation
Tielemans et al. [99]	2015	Netherlands	Community	RCT	USER-P	*n* = 113	Baseline	Postintervention	Postintervention (3 months)	Postintervention (9 months)	Researcher/research assistant	Self-management 55.2 (8.9), education 58.8 (8.7)	Operational
van Mierlo et al. [100]^∗^	2016	Netherlands	Community	Longitudinal cohort study	USER-P	*n* = 368	Poststroke (2 months)	Poststroke (6 months)	Poststroke (12 months)	Poststroke (24 months)	Researcher/research assistant	66.8 (12.3)	Operational
Vincent-Onabajo et al. [101]	2014	Nigeria	Research laboratory and community	Case series	LHS	*n* = 83	Poststroke (1 month)	Poststroke (3 months)	Poststroke (6 months)	Poststroke (9 months and 12 months)	Not described	Male 60.7 (12.4), female 58.1 (12.6)	ICF
Viscogliosi et al. [102]^∗^	2011	Canada	Inpatient and community	Comparative study no controls	LIFE-H	*n* = 197	Poststroke (3 months)	Poststroke (6 months)	Poststroke (9 months)		OT	76.9 (7.0)	DCP
Worrall et al. [103]	2017	Australia	Inpatient and community	Prospective longitudinal cohort study	ALA	*n* = 58	Poststroke (3 months)	Poststroke (6 months)	Poststroke (9 months)	Poststroke (12 months)	Not described	66.1 (13.6)	ICF
Yang and Kong [104]^∗^	2013	Singapore	Inpatient	Prospective observational cohort study	SF-36	*n* = 122	Baseline	Predischarge			OT, PT	58.2 (10.5)	Operational

Note: ADL: activity of daily living; ALA: assessment for living with aphasia; DCP: disability creation process; FAI: Frenchay activity index; GAS: goal attainment scale; GPS: global positioning system; ICF: International Classification of Functioning, Disability and Health; IMPACT-P: participation subsection of the ICF measure of participation and activities; IPA: impact on participation and autonomy; LHS: London handicap scale; LIFE-H: assessment of life habits; MAL: motor activity log; MPAI-4: Mayo-Portland adaptability inventory; mRS: modified ranking scale; NEADL: Nottingham extended activities of daily living; NLQ: Nottingham leisure questionnaire; OGQ: occupational gaps questionnaire; PASIPD: physical activity scale for individuals with physical disabilities; RAND-36: physical function index of the medical outcomes study RND-36 item health survey; RCT: randomized control trial; RNL: reintegration of normal living; RTP: repetitive task practice; SF-36: short form 36; SIS-P: stroke impact scale participation domain; USER-P: Utrecht scale for evaluation of rehabilitation-participation; VR: virtual reality. ^∗^Cohort studies that statistically tested for changes in participation.

**Table 3:** Time point of participation measurement by authors measuring participation longitudinally after stroke.

Poststroke	Poststroke	Postintervention	Postbaseline	Postdischarge
At baseline			34	
Pre/at discharge				3
Immediately		14		
5 days	1			
1 week		1	1	
2 weeks	1		1	
1 month	6	1	2	
6 weeks	1	3		
2 months	4	3	2	
10 weeks			1	
3 months	15	6	4	1
4 months	2	1	1	
5 months		2		
6 months	18	7	3	2
30 weeks		1		
8 months		1		
9 months	6		1	
12 months	13	4	1	2
18 months	2			
24 months	1			
2–4 years	2			

**Table 4:** Summary of randomized control trial data in this review on longitudinal participation outcomes after stroke.

Author	Year	Country	Setting	Intervention	Age (*yr*) mean (SD), median (IQR)	Sex (% male)	Time poststroke (*months*) mean (SD) or median [range]	Association on participation
Bertilsson et al. [49]	2016	Sweden	Inpatient and community	Client-centred ADL intervention specifically guided by client needs and expressed desires, focused on enabling the person with stroke to become an active agent in daily activities and participation in everyday life, and the caregivers were invited to participate in rehabilitation as much as they wanted.	Client-centred 74.1 (9.5), usual 71.3 (10.1)	Client-centred 53%, usual care 62%	Not described	There was no significant difference between those receiving client-centred ADL intervention and usual care in terms of participation at 12 months.
Combs-Miller et al. [53]	2014	USA	Research laboratory and community	Comparison of two types of walking training: body weight-supported and overground.	Body weight-supported 56.20 (7.61), overground walking 65.50 (6.17)	Body weight-supported 40%, overground walking 70%	Body weight-supported 62.3 (48.6), overground walking 60.0 (51.7)	No evidence found to support this type of intervention (body weight-supported or overground walking training) on improving participation.
Desrosiers et al. [56]	2007	Canada	Community	Leisure education program at home once a week for 8–12 weeks. Control participants (*n* = 29) were visited at home at a similar frequency.	Intervention 61 (5), control 60 (5)	Intervention 16 (57.1), control 12 (42.9)	Months: experimental 24.5 (25.7), control 32.7 (37.8)	Some evidence to support the use of this leisure education program for improving the number of minutes of leisure and number of leisure activities participated in compared to control group.
Flansbjer et al. [62]	2008	Sweden	Community	Progressive resistance training on muscle strength, muscle tone, gait performance, and perceived participation after stroke.	Intervention 61 (5), control 60 (5)	Intervention 60%, control 56%	Baseline: intervention 18.9 (7.9), control 20 (11.6)	Some evidence to support this type of intervention (supervised progressive resistance training of the knee extensors and flexors) compared to usual care on improving participation after the intervention and maintained at 5 months.
Guidetti et al. [64]	2015	Sweden	Community	The CADL intervention was conducted within a client-centred context. The UADL interventions varied in extent and methods according to the knowledge and clinical experience of the individual OT and according to the routines and praxis of the participating rehabilitation units.	CADL 74 (10), UADL 71 (11)	CADL 57%, UADL 63%	CADL 25 [6–96], UADL 28 [3–115]	There were no differences between the groups regarding changes in perceived participation, independence in ADL, or life satisfaction during the first 12 months. There was a trend towards a clinically meaningful positive change in perceived participation that favoured client-centred ADL intervention. Good design.
Kluding et al. [71]	2013	USA	Community	Standard treatment versus electric stimulation therapy to improve foot drop.	Interventions 60.7 (12.2), control 61.6 (11.0)	Intervention 56.8%, control 43.2%	Intervention 4.8 (5.3) *yrs*, control 4.3 (4.1) *yrs*	No difference in participation between the intervention of 30 weeks of either foot drop stimulator or a standard ankle foot orthosis.
Kootker et al. [72]	2017	Netherlands	Community	Individually tailored CBT for reducing depressive symptoms.	CBT 61 (45–79), CCT 61 (25–76)	CBT 61.3%, CCT 63.3%	CBT 26 [2–243], CCT 21.5 [2–138]	Some evidence to support the use of both CBT and CCT to improve participation at this level of intervention.
Kutner et al. [73]	2010	USA	Inpatient and community	This preliminary study explored change in patient-reported, health-related quality of life associated with robotic-assisted therapy combined with reduced therapist-supervised training. Sixty hours of therapist-supervised repetitive task practice (RTP) was compared with 30 hours of RTP combined with 30 hours of robotic-assisted therapy.	RTP 51.0 (11.3), combined therapy group 61.9 (13.4)	Total 59%, RTP 71%, combined therapy group 50%	Total *days* 234.4 (121.8), RTP *days* 184.1 (126.5), combined therapy group *days* 269.6 (111.1)	Significant differences in participation pre- and postintervention for RTP group at 2 months follow-up but not for combined therapy group.
Lund et al. [78]	2011	Norway	Community	A lifestyle course in combination with physical activity (intervention group) compared with physical activity alone (control group). Both programmes were held once a week for nine months.	Intervention 75 (7.2), control 79 (6.5)	Intervention, control 43%	Intervention 161 (178) *days*, control 137 (124) *days*	No statistically significant differences between the groups at the nine-month follow-up.
Mayo et al. [82]	2013	Canada	Community	Two dose-equivalent interventions, one involving stationary cycling and the other disability-targeted intervention, were tested. Both protocols required daily moderate intensity exercise at home building up to 30 minutes per day. One group exercised on a stationary bicycle; the second group carried out mobility exercises and brisk walking. An observer-blinded, randomized, pragmatic, trial with repeated measures. At baseline and after 1, 6, and 12 months of exercise and home-based assessments at 3 and 9 months.	Cycle 67.7 (14.4), exercise 67.8 (12.3)	Cycle 80%, exercise 59%	Cycle *days* 265.4 (131.8), exercise *days* 252.0 (165.3)	A significant effect for role participation was found in the exercise group for cycling versus exercise.
Pang et al. [86]	2005	Canada	Community	19 weeks (1-hour sessions, 3 sessions per week). Intervention included the Fitness and Mobility Exercise (FAME) program 10 minutes initially, with increment of 5 minutes every week, up to 30 minutes of continuous exercise as tolerated.	Intervention group 65.8 (9.1), control 64.7 (8.4)	79%	Intervention *yrs* 5.2 (5.0), control *yrs* 5.1 (3.6)	There was no significant time × group interaction on participation.
Parker et al. [87]	2001	United Kingdom	Community	Occupational therapy interventions at home for up to six months after recruitment, minimum of 10 sessions lasting not less than 30 minutes each. The treatment goals set in the ADL group were in terms of improving independence in self-care tasks, and therefore, treatment involved practicing these tasks (such as preparing a meal or walking outdoors). For the leisure group, goals were set in terms of leisure activity, and so, interventions included practicing the leisure tasks as well as any ADL tasks necessary to achieve the leisure objective.	Leisure 72 (65–79), ADL 71 (66–78), control 72 (65–78)	Leisure 58%, ADL 62%, control 54%	Not described	At six months and compared to the control group, those allocated to leisure therapy had nonsignificantly better leisure participation scores. Those allocated to the ADL group had nonsignificantly worse leisure scores compared to controls. The results were similar at 12 months.
Rochette et al. [91]	2013	Canada	Community	YOU CALL participants were provided with the name and phone number of a trained healthcare professional whom they were free to contact should they feel the need. WE CALL participants received a multimodal support intervention including new or ongoing issues, family functioning, and individualized risk factors. Call frequency was weekly for the first 2 months, biweekly during the third month, and monthly for the past 3 months and included support material and referrals as needed.	YOU CALL 63.2 (12.4), WE CALL 61.7 (12.7)	YOU CALL 53.2%, WE CALL 62%	Not described	No significant differences were seen between groups at 6 months. Significant improvements in social participation for both groups from 6 to 1 year. No significance between group differences.
Sabariego et al. [92]	2013	Germany	Inpatient and community	ICF-based patient-education programme. The programme was performed by a psychologist in 1-hr sessions over 5 days. The group size was four participants, and it was a closed group.	Intervention 55.3 (12.6), control 59.3 (12.7)	Intervention 63%, control 45%	Intervention *days* 151.1 (399.3), control *days* 149.5 (634.7)	Participation improved for both groups, but no between-group difference was found. Large study, good design. Exploratory post hoc model identified life satisfaction, self-efficacy, memory, and mood as significant factors for change with SIS-P as dependent variable.
Sandberg et al. [93]	2016	Sweden	Community	Sixty minutes of group aerobic exercise, including 2 sets of 8 minutes of exercise with intensity up to exertion level 14 or 15 of 20 on the Borg rating of perceived exertion scale, twice weekly for 12 weeks.	Intervention 71.3 (7.0), control 70.4 (8.1)	50%	Intervention *days* 4.9 (5.8), control *days* 6.3 (7.3)	Significant change in SIS-P from preintervention to postintervention (aerobic exercise versus no therapy); also, significant time effect within groups but nonsignificant group × time effect and nonsignificant between-subjects' effects.
Studenski et al. [97]	2005	USA	Community	The 36-session, 12-week, home-based exercise program, supervised by an occupational or physical therapist, targeted strength (major muscle groups of the upper and lower extremity using elastic bands and body weight), balance, and endurance (using an exercise bicycle) and encouraged use of the affected upper extremity. There were structured protocols for the exercise tasks, criteria for progression, and guidelines for reintroducing therapy after intercurrent illness. After completing the intervention, participants received written guidelines for continued exercise.	Intervention 68.5 (9.0), usual care 70.4 (11.3)	53%	Intervention *days* 77.5 (28.7), usual care *days* 74.1 (27.2)	Support for this intervention (home-based exercise program) compared to usual care immediately after the intervention but not at 6-month follow-up.
Tielemans et al. [99]	2015	Netherlands	Community	The 10-week self-management intervention consisted of 7 sessions, 6 × 2 h sessions in the first 6 weeks and 1 × 2 h booster session in week 10. It was provided to groups of 4–8 participants by 2 rehabilitation professionals (e.g., psychologist or occupational therapist) at hospitals and rehabilitation centre outpatient facilities. The intervention aimed to teach proactive action planning strategies within 4 themes: “handling negative emotions,” “social relations and support,” “participation in society,” and “less visible stroke consequences.” The 10-week education intervention consisted of 3 × 1 h sessions in the first 6 weeks and 1 × 1 h booster session in week 10. It was provided in groups of 4–8 participants by one rehabilitation professional at hospital and rehabilitation centre outpatient facilities.	Self-management 55.2 (8.9), education 58.8 (8.7)	Self-management 54.8%, education 60%	Self-management 15.6 (20.9), education 21.9 (34.1)	No significant differences between self-management and education intervention, on either primary or secondary outcome measures, but there were trends towards a difference in participation restriction at follow-up.

Note. ADL: activity of daily living; CBT: client-centred therapy; CBT: cognitive behavioral therapy; ICF: International Classification of Functioning, Disability and Health; RTP: repetitive task practice.

**Table 5:** Tools measuring participation longitudinally after stroke.

Participation measure	Frequency of participation measures
SIS-P	24
LIFE-H	5
LHS	4
USER-P	3
SF-36	3
RNL	2
FAI	2
MPAI-4	1
SIS-P, NEADL	1
GPS	1
ALA	1
Number of minutes	1
NLQ	1
SIS-P, OGQ	1
SIS-P, RAND	1
NEADL, mRS	1
GAS	1
PASIPD	2
FAI, 6-minute walk test	1
IMPACT-P	1
IPA	1
MAL	1
Grand total	59

Note. ALA: assessment for living with aphasia; FAI: Frenchay activity index; GAS: goal attainment scale; GPS: global positioning system; IMPACT-P: participation subsection of the ICF measure of participation and activities; IPA: impact on participation and autonomy; LHS: London handicap scale; LIFE-H: assessment of life habits; MAL: motor activity log; MPAI-4: Mayo-Portland adaptability inventory; mRS: modified ranking scale; NEADL: Nottingham extended activities of daily living; NLQ: Nottingham leisure questionnaire; OGQ: occupational gaps questionnaire; PASIPD: physical activity scale for individuals with physical disabilities; RAND-36: physical function index of the medical outcomes study RND-36 item health survey; RNL: reintegration of normal living; SF-36: short form 36; SIS-P: stroke impact scale participation domain; USER-P: Utrecht scale for evaluation of rehabilitation-participation.

**Table 6:** Definitions of participation reported by authors measuring participation longitudinally after stroke.

Definition of participation	Frequency
Operational definitions	24
ICF	20
LIFE-H	6
Meaningful activities/occupations	2
Social participation	2
ICF and role participation	1
Self-perceived participation	1
ICF and meaningful activities/occupations	1
Community participation (role contribution)	1
Role participation	1
Total	59

Note. ICF: International Classification of Functioning, Disability and Health; LIFE-H: assessment of life habits.

**Table 7:** Definitions of participation relative to the proportion of studies from each continent in this review on longitudinal participation outcomes after stroke.

	North America	Europe	Australasia	South America	Africa	Mixed
Operational definitions	7	12	3	1		1
ICF	9	5	3		2	1
LIFE-H	6					
Meaningful activities/occupations	1		1			
Social participation	1		1			
Community participation (role contribution)	1					
Role participation	1					
ICF and role participation	1					
Self-perceived participation		1				
ICF and meaningful activities/occupations			1			
Total	26	19	9	1	2	2

Note. ICF: International Classification of Functioning, Disability and Health; LIFE-H: assessment of life habits.
